# Human and camel cystic echinococcosis – a polyclonal antibody-based sandwich ELISA for its serodiagnosis with molecular identification

**DOI:** 10.1007/s11259-024-10375-3

**Published:** 2024-04-26

**Authors:** A. Maher, N. I. Toaleb, R. M. Shaapan, D. Aboelsoued, M. B. Salman, S. Zaky

**Affiliations:** 1 Department of Zoonotic Diseases, Veterinary Research Institute, National Research Centre, Dokki, Giza Egypt; 2 Department of Parasitology and Animal Diseases, Veterinary Research Institute, National Research Centre, Dokki, Giza Egypt; 3 Hepato-Gastroenterology and Infectious Diseases Department, Faculty of Medicine, Al-Azhar University, Cairo, Egypt

**Keywords:** Echinococcus granulosus, PCR, Protein-A sepharose gel affinity chromatography, SDS-page, Sandwich ELISA, Indirect ELISA

## Abstract

Cystic echinococcosis (CE) is an emergent neglected disease affecting human and animals in Egypt with a wide distribution and incidence. This study aimed to evaluate the use of a polyclonal antibody-based sandwich ELISA in the detection of *Echinococcus granulosus* antigen in human and camel sera. Hydatid cyst protoscoleces antigen (PsAg) was isolated from hydatid cysts collected from naturally infected camel livers and lungs. PsAg was used for immunization of rabbits to raise IgG polyclonal antibodies (IgG PsAb). IgG PsAb were then precipitated, purified using Protein-A Sepharose gel and labeled with horseradish peroxidase enzyme. We assayed the purity of the IgG PsAb, and the two prepared *E. granulosus* antigens CPsAg from camel cysts and HPsAg from human cysts by Sodium dodecyl-sulfate polyacrylamide gel electrophoresis (SDS-PAGE). The resulted protein bands of the prepared CPsAg appeared at different molecular weights: 180, 90, 68, 54, 42 and 22 kDa while, HPsAg shared with it in 4 common bands at 68, 54, 42, and 22 kDa. The purified IgG PsAb had been resolved at two bands at 52 kDa and at 32 kDa. Sandwich ELISA were performed for the detection of circulating *E. granulosus* antigens in sera of human (*n* = 183) and camels (*n* = 190). The purified IgG PsAb showed strong reactivity against *E. granulosus* infected human and camel samples and no cross reactivity neither with free-healthy negative sera nor with others parasitic diseases (Schistosomiasis, Fascioliasis, Toxoplasmosis, Ancylostomiasis for human samples and Fascioliasis, ticks’ infestation, Eimeriosis, Cryptosporidiosis, Nasal myiasis, Toxoplasmosis for camel samples). The sensitivity of the assay was 98.25% (56/57) and 96.9% (31/32) against human and camel samples, respectively. Specificity was 100% in both human and camel samples. Sandwich ELISA detected CE in 33.3% (24/72) and 55.6% (50/90) random human and camel samples, respectively. Indirect ELISA, using CPsAg, was used for detection of antibodies in positive human and camels’ sera and detected 96.5% (55/57) and 93.8% (30/32) of human and camel samples, respectively. In our study, Genomic DNA was extracted from protoscoleces fluid of human liver hydatid cysts to identify the *Echinococcus* sp. isolate based on NADH dehydrogenase subunit 1 (NAD1) gene by Polymerase Chain Reaction (PCR) and the isolate (GenBank: OP785689.1) were identified as *E. granulosus sensu lato* genotype. In conclusion, Sandwich ELISA technique was found to be a potent and sensitive assay for detection of hydatid antigen in both human and camel samples.

**Supplementary Information:**

The online version contains supplementary material available at 10.1007/s11259-024-10375-3.

## Introduction

Cystic echinococcosis (CE) is important helminthic zoonotic disease caused by the infection with *Echinococcus granulosus* belonging to the family Taeniidae **(**Thompson 2017; Ebrahimipour et al. 2019; Zhang et al. 2023). Fecal materials of *E. granulosus*-infected dogs and canids (definitive hosts) contaminate the environment spreading infective eggs which are taken by fecal-oral route in humans and animals (intermediate hosts) **(**King and Fairley 2015**)** causing significant morbidity and mortality in humans as well as significant economic losses in livestock industry worldwide **(**Nigo et al. 2022; Cai et al. 2023). In livestock, CE could result in reduced birth rate, low yield and quality of animal meat and wool, delayed growth, destructed viscera, organ condemnation, and even death **(**Benner et al. 2010; Cai et al. 2023). In humans, economic losses are resulted from increased surgery costs, hospital care, and impaired labor productivity **(**Singh et al. 2014; Sen et al. 2019). Its prognosis depends on cyst number, stage, and location making control of this disease complex **(**Ali et al. 2020). The high prevalence of CE in humans and animals is usually observed in temperate regions (Mediterranean regions, North and East Africa, and Central Asia) **(**Grosso et al. 2012).

Cysts are fluid-filled vesicles with two layers. The outermost laminated layer which is an acellular coat secreted by the inner germinal layer. The inner germinal layer is the cellular proliferative sheet of the hydatid cyst which encloses brood capsules, protoscoleces **(**Pedrosa et al. 2000), undifferentiated cells, storage cells, and muscle cells (Siles-Lucas et al. 2017). Due to absence of specific clinical signs and symptoms, CE is commonly performed at necropsy in post-mortem (PM) examination in animals **(**Craig et al. 2015) and by laboratory techniques and radiological imaging in humans **(**Alli et al. 2011). Ultrasonography, computerized tomography, and serology are useful diagnostic tools for human hydatidosis, and the disease is considered a public health challenge and needs accurate differential diagnosis from any cystic mass/s in the abdomen (Elaadli et al. 2022). Immunodiagnostic laboratory techniques are widely used to confirm CE diagnosis in suspected cases or follow up after surgical and chemical treatments, especially Enzyme Linked Immunosorbent Assay (ELISA) and Indirect hemagglutination test (IHAT) **(**Zhang et al. 2012).

ELISA is a fast, easy and non-expensive test to perform with high sensitivity and specificity **(**Hassanain et al. 2021). The main obstacle of antibody tests is that they couldn’t distinguish between past and present infections. Antigen detection techniques might help to avoid this problem (Doiz et al. 2001) and might be useful in the immunodiagnosis of hydatid disease (Toaleb et al. 2023). Circulating hydatid antigens are detected in the serum only during active infection, and their levels decrease gradually after surgical removal of the cyst or successful chemotherapy **(**Devi and Parija 2003; Sadjjadi et al. 2009; Bauomi et al. 2015). Also, antigen detection assays could help in assessing the efficacy of treatment, especially after surgical removal of hydatid cyst **(**Bauomi et al. 2015).

Because of the importance of cystic echinococcosis in human and animal health, rapid and precise diagnostic methods are urgently needed (Hassanain et al. 2021). Consequently, the current research aims to evaluate a specific and simple diagnostic efficacy of purified polyclonal antibody IgG raised against *E. granulosus* protoscoleces antigen (PsAg) for detection circulating *E. granulosus* antigen in humans and animal’s sera using Sandwich ELISA and compared it with antibody detection method.

## Materials and methods

### Human patients and study area

This cross-sectional study was carried out during the period from December 2021 to October 2023 on 183 patients who attended in the Gastroenterology Outpatient Clinics, Internal Medicine and Surgery Departments, Al-Azhar Teaching Hospital and Al-Kasr Al-Ainy School of Medicine, Cairo University, Cairo, Egypt. An informed written consents were obtained from patients who participated in this study.

### Animals and study area

One hundred ninety blood samples were collected from camels through several visits to main local abattoirs of Giza (Nahia, El-warrak and El-moneb) and Cairo (EL-Basatin abattoir), Egypt, during the period December 2021 to October 2023.

## Blood samples

### Human blood samples

One hundred eighty-three blood samples were collected from patients who complained about digestive disturbances, fever, and abdominal pain. These blood samples were divided into four groups:

I **17** blood samples collected from apparently healthy individuals free from intestinal parasites represented as negative controls.
II **57** blood samples represented the positive controls including **53** blood samples from individuals that were diagnosed to have CE in their livers, chest and other organs as proved by sonography, Computed Tomography (CT) and Magnetic Resonance Imaging (MRI), and **4** blood samples collected from individuals that had CE in their livers as proved by sonography, CT, and MRI to have CE with surgical confirmation and removal. Cysts removed during surgery were confirmed to be hydatid cysts with the presence of fertile protoscoleces in the aspirated cysts’ fluid. Sera from these patients were collected before surgery on admission to surgical ward (gold standard positive control).
III **72** blood samples were randomly obtained.
IV **37** blood samples were collected from individuals that were infected with other parasites [**10***Schistosoma mansoni* (schistosomiasis), **7***Fasciola gigantica* (fascioliasis), **15***Toxoplasma gondii* (toxoplasmosis), and **5***Ancylostoma duodenale* (Ancylostomiasis)].

All blood samples were collected from all cases, sera were separated, aliquoted and kept at − 20 °C until used.

### Camel blood samples

One hundred ninety blood samples were collected from camels through several visits to main local abattoirs during veterinary medical examinations:

I **32** blood samples from camels that were infected by *E. granulosus* collected at postmortem inspection (PM; gold standard positive control sera).
II **15** blood samples collected from healthy young camels, free of cysts and other parasitic infections as confirmed by PM inspection and fecal examination (gold standard negative control sera).
III **53** blood samples from camels that were positive for other parasites (**5** infected with *F. gigantica* (Fascioliasis), **12** blood samples from camels infested with *Hyalomma dromedarii* ticks, **6** samples from camels with Coccidiosis (Eimeriosis) as proved by fecal examination, **10** samples from camels infected with *Cryptosporidium parvum* (Cryptosporidiosis) as proved by fecal examination, **13** samples from camels infested with *Cephalopina titillator* larvae as shown after examination of slaughtered camels’ skulls and **7** samples from camels that were positive to *Toxoplasma* as proven by ELISA).
IV **90** blood samples were randomly obtained.
  All blood samples were collected from all cases, sera were separated, aliquoted and kept at − 20 °C until used.

## Parasite

### Human hydatid cysts

Four human hydatid cysts were recovered from postoperative patients admitted to the Internal Medicine and Surgery Departments, Al-Azhar Teaching Hospital and Al-Kasr Al-Ainy School of Medicine, Cairo University, Cairo, Egypt. Protoscoleces were aspirated from cysts, pooled, and washed with sterile normal saline. In our laboratory, every sample was examined by light microscope (CX41 Olympus Microscope, Olympus Corporation, Japan) and 0.1% eosin (Sigma-Aldrich, Louis, MO, USA) staining to assess the cyst fertility and viability (Daryani et al. 2007). Cyst fluids were centrifuged at 5000 rpm for 30 min and each sample was stored at − 20 °C until being used for the molecular analysis (Piccoli et al. 2013).

### Camel hydatid cysts

Thirty-two hydatid cysts (12 large fertile hydatid cysts from infected livers and other 20 large fertile cysts from infected lungs) were collected from slaughtered camels during PM inspection. Hydatid cysts were placed in sterile saline solution and transported to the laboratory in ice box. Cyst contents were aseptically aspirated, centrifuged at 5000 rpm for 30 min, and examined for the presence of protoscoleces. The precipitates of the fertile cysts were stained with 0.1% eosin solution for 10 min and the viability of the protoscoleces was examined under a light microscope (Bahmani and Anisian 2020**).** Then, protoscoleces were collected from fertile cysts of liver, collected protoscoleces were washed several times in sterile normal saline (Dyab et al. 2017).

### DNA extraction

The Genomic DNA was extracted from protoscoleces fluid of four fertile liver human hydatid cysts using the QIAamp DNA mini kit (Cat. No. 51,304, QIAGEN, Germany) according to manufacturer’s protocol. The DNA concentrations were estimated by Q9000 microvolume spectrophotometer (Quawell, USA) and stored at − 20 °C.

### Polymerase chain reaction (PCR) and electrophoresis

PCR was performed on the extracted DNA using primers which target 500 bp fragment of NADH dehydrogenase subunit 1 (NAD1) gene (F: 5ʹ- AGA TTC GTA AGG GGC CTA ATA − 3ʹ and R: 5ʹ- ACC ACT AAC TAA TTC ACT TTC − 3ʹ) according to Aboelhadid et al. (2013) using T100 Thermal Cycler (BIO-RAD, Singapore). The PCR products were then visualized by Gel Doc™ XR + Molecular Imager (BIO RAD, California, USA) in agarose gel electrophoresis (1.5%), stained by RedSafe (Intron Biotechnology, Republic of Korea), and measured by a 100 bp ladder (Cat. No. 239,035, QIAGEN, USA). Negative and positive controls were involved in the PCR protocol.

### Sequencing and phylogenetic analysis

After PCR, products of the most intense bands on gel electrophoresis were purified by a Gel Extraction Kit (Cat. No. NA006-0100, GeneDirex, Taiwan) according to manufacturer’s instructions. The purified products were sequenced by ABI 3130 Automated Sequencer (Applied Biosystems, USA) using Big Dye Terminator v3.1 Cycle Sequencing Kit (Applied Biosystems, USA). Obtained sequences were corrected using ChromasPro 1.7 software (Technelysium Pty Ltd., Australia), compared by BLASTn (https://blast.ncbi.nlm.nih.gov/Blast.cgi) with previous sequences available in GenBank and submitted to GenBank. Then, multiple sequence alignments were performed using CLUSTAL W v1.83, in the MegAlign module of the Laser gene software package (DNASTAR, USA). Phylogenetic analysis was performed using MEGA6 software (Tamura et al. 2013).

### Preparation of protoscoleces antigens

Human and camels (the used camel isolate was previously identified as GenBank: OQ443068.1; Toaleb et al. 2023) protoscoleces’ antigens (HPsAg and CPsAg) were prepared. Briefly, the hydatid cyst fluid (HCF) was obtained by aseptic puncture from fertile hydatid cysts of camel liver origin, centrifuged at 6000 rpm for 45 min at 4ºC, and the sediments containing protoscoleces were collected in sterile tubes. The protoscoleces were washed three times with Phosphate Buffered Saline (PBS) pH = 7.2, subjected to several freezing and thawing cycles and then sonicated using a 150w ultrasonic disintegrator (10 cycles/12 sec/60 Hz frequency) until no intact protoscoleces were visible microscopically. Protoscoleces mixture was centrifuged at 16,000 rpm for 30 min. The supernatant was collected, aliquoted, and stored at − 20 °C **(**Carmena et al. 2005). The protein content of the two prepared antigens was estimated by Lowry’s method **(**Lowry et al. 1951).

### Rabbit polyclonal antibody IgG (anti-hydatid IgG)

We used five healthy 3 months aged New Zealand rabbits weighing 2–2.5 Kg obtained from local market. They were daily examined for 2 weeks to ensure they are parasite-free. Rabbit hyperimmune serum was raised against CPsAg as described by Guobadia and Fagbemi (1997**)** and Fagbemi et al. (1995). Briefly, 40 µg/Kg of CPsAg was mixed with same volume of Freund’s complete adjuvant (Sigma-Aldrich, Cat No. F5881) and subcutaneously injected into each rabbit. After 2 weeks, a booster dose of CPsAg (40 µg/Kg) with an equal volume of Freund’s incomplete adjuvant (Sigma-Aldrich, Cat No. F5506) was injected subcutaneously into each rabbit. Second and third booster doses were injected at the 21st and 28th days, respectively. Blood samples were taken by ear vein bleeding 4 days after last injection and monitored for the production of antibodies IgG to CPsAg by indirect ELISA according to Engvall and Perlmann (1971**).**

### Purification of polyclonal antibody IgG

Anti-hydatid IgG (IgG PsAb) was precipitated using 50% Ammonium sulphate solution according to Wingfield (2016). Then, dialysis IgG PsAb against 0.15 M PBS for 3 days at 4 °C was performed. Polyethylene glycol was used to concentrate the IgG PsAb. Furthermore, purification of IgG PsAb was performed with Affinity chromatography on Protein-A Sepharose gel (Sigma, St. Louis, Missouri, USA) according to Abd El Hafez et al. (2010) and Toaleb et al. (2023). The protein content of IgG PsAb was estimated following the method of Lowry et al. (1951). The reactivity of Anti-hydatid IgG (IgG PsAb) against *Echinococcus* antigen (PsAg) was measured by indirect ELISA.

### Labeling of antibodies (IgG PsAb) by horseradish peroxidase (HRP)

Conjugation of IgG PsAb with HRP was performed as previously described by Avrameas (1969**)** and Toaleb et al. (2023). In brief, HRP enzyme (10 mg) was mixed with IgG PsAb (5 mg) in 1 mL of 0.1 M phosphate buffer (pH = 6.8). Then they were dialyzed at 4 ◦C overnight against 0.1 M PBS (pH = 6.8). After that, 50 µL diluted glutaraldehyde was added to the mixture with gentle shaking for 3 h at room temperature. Glycine solution (2 M) was added, left for 2 h at room temperature and then dialyzed overnight again. The mixture was centrifuged at 10,000 ×*g* for 30 min at 4 ◦C and the supernatant was aspirated to sterile tubes and one volume of glycerol was added. The prepared labeled IgG PsAb was stored at − 20 ◦C until use.

### Sodium dodecyl-sulfate polyacrylamide gel electrophoresis (SDS-PAGE)

Electrophoresis of IgG PsAb, CPsAg and HPsAg was performed in 10% polyacrylamide gels according to method described by Laemmli (1970). Each protein of IgG PsAb, CPsAg and HPsAg was mixed with sample buffer containing 2-mercaptoethanol before loading to the gel, separately. After separation, slab gel was stained with Coomassie Brilliant Blue dye. Relative molecular weights of bands were calculated using prestained protein marker (GeneDirex BLUltra, USA). Molecular weights were analyzed using Bio-Rad Imager (Gel Doc™ XR^+^, California, USA).

### A polyclonal antibody-based sandwich ELISA

Sandwich ELISA was performed following the method of Toaleb et al. (2023). In brief, 4 µg/mL of rabbit IgG PsAb was diluted in coating buffer (dilution 1:25), added into 96-well ELISA plates (100 µL/well), and incubated at 4 ◦C overnight. The plates were washed 3 times by PBS-Tween-20 (0.1 MPBS, pH = 7.4, + 0.05% Tween 20) to remove excess antibodies. Wells were blocked with blocking buffer (1% bovine serum albumin, BSA, 200 µL/well), incubated at 37 ◦C for 2 h, and then washed 3 times with PBS-Tween-20. Triplicates of human and camel sera (diluted 1:100 with PBS; 100 µL/well) were added and the plates were incubated at 37 ◦C for 2 h. Diluted HRP-conjugated IgG PsAb (1:100; 100 µL/well) were added after another washing step, and the plates were incubated at 37 ◦C for 90 min then washed 3 times with washing buffer. The reaction was visualized by adding 100 µl/well of Ortho-phenylenediamine (OPD, Sigma-Aldrich) substrate solution for 20–30 min in the dark at room temperature. Reaction was stopped by 50 µl/well of 0.1M H_2_SO_4_. Optical densities (OD) were read at 450 nm with an ELISA microplate reader (Bio-Tek Instruments, Winooski, VT, USA). Positive and negative controls were used at the same plate and all samples and controls were assayed in triplicates. The cut-off point was obtained by mean values of OD of negative control plus three times the standard deviation (SD) according to Jin et al. (2013). Diagnostic sensitivity, specificity, diagnostic efficacy as well as positive and negative predictive values were calculated according to Parikh et al. (2008) and as described by Toaleb et al. (2023).

### Indirect ELISA

Indirect ELISA, using CPsAg, was performed for detection of antibodies in human and camels’ sera as described in Hassanain et al. (2021). In Brief, an ELISA plate was coated with CPsAg (4 µg/mL) overnight, washed 3 times with washing buffer and blocked by BSA 1%. Two-fold serial dilutions of the purified IgG CPsAb were added into wells. The plate was washed and the conjugate (Anti-rabbit IgG HRP, Sigma Chemical Co., St. Louis, MO, USA) was used. The OPD substrate (Sigma-Aldrich) was used for color development in the reaction. OD were measured at 450 nm with EL×800UV BioTek microplate reader (Bio-Tek Instruments, Winooski, VT, USA).

### Statistical analysis

Statistical analyses were performed by SPSS (version 19.0 for Windows, IBM Corp., USA). Data of CE infection rates, and Sandwich ELISA were analyzed using Chi square test. *P* < 0.05 was considered statistically significant. The diagnostic accuracy parameters of the test were evaluated by calculating the sensitivity, specificity, area under the curve (AUC), and receiver operating characteristic (ROC) curve, Chi square using SPSS software.

## Results

### The cysts fertility and viability

Of the total hydatid cysts of camel’s lungs and Livers examined, fertile cysts accounted for all 32 camels’ cysts were 68.75% (22/32 cysts). We found 91.7% (11/12) fertile and 8.3% (1/12) calcified cysts in camel liver hydatid cysts examined. Whereas, in camel lung hydatid cysts, 60% (12/20) were fertile, 10% (2/20) were calcified and 30% (6/20) were sterile lung cysts. Average viability of fertile cyst protoscoleces was obtained (90.9 ± 0.8) in camel liver cysts and (58.3 ± 1.7) in camel lung cysts, with a statistically significant difference (*P* < 0.05). In human the highest viability of fertile cyst protoscoleces was obtained in cysts removed from human during surgery (in the aspirated cyst fluid) as shown in Table 1.

**Table 1:** Determination of fertility and viability of protoscoleces of hydatid cysts in humans under surgery and slaughtered camels based on organ type

Species	Organ	Type of Cysts			
		Fertile % (n)	Sterile % (n)	Calcified % (n)	Total % (n)
Human	Liver	100% (4/4)	------	-------	100% (4)
Camel	Liver	91.7% (11/12)	------	8.3% (1/12)	100% (12)
	Lung	60% (12/20)	30% (6/20)	10% (2/20)	100% (22)

### PCR and phylogenetic analysis

**Fig. 1:**
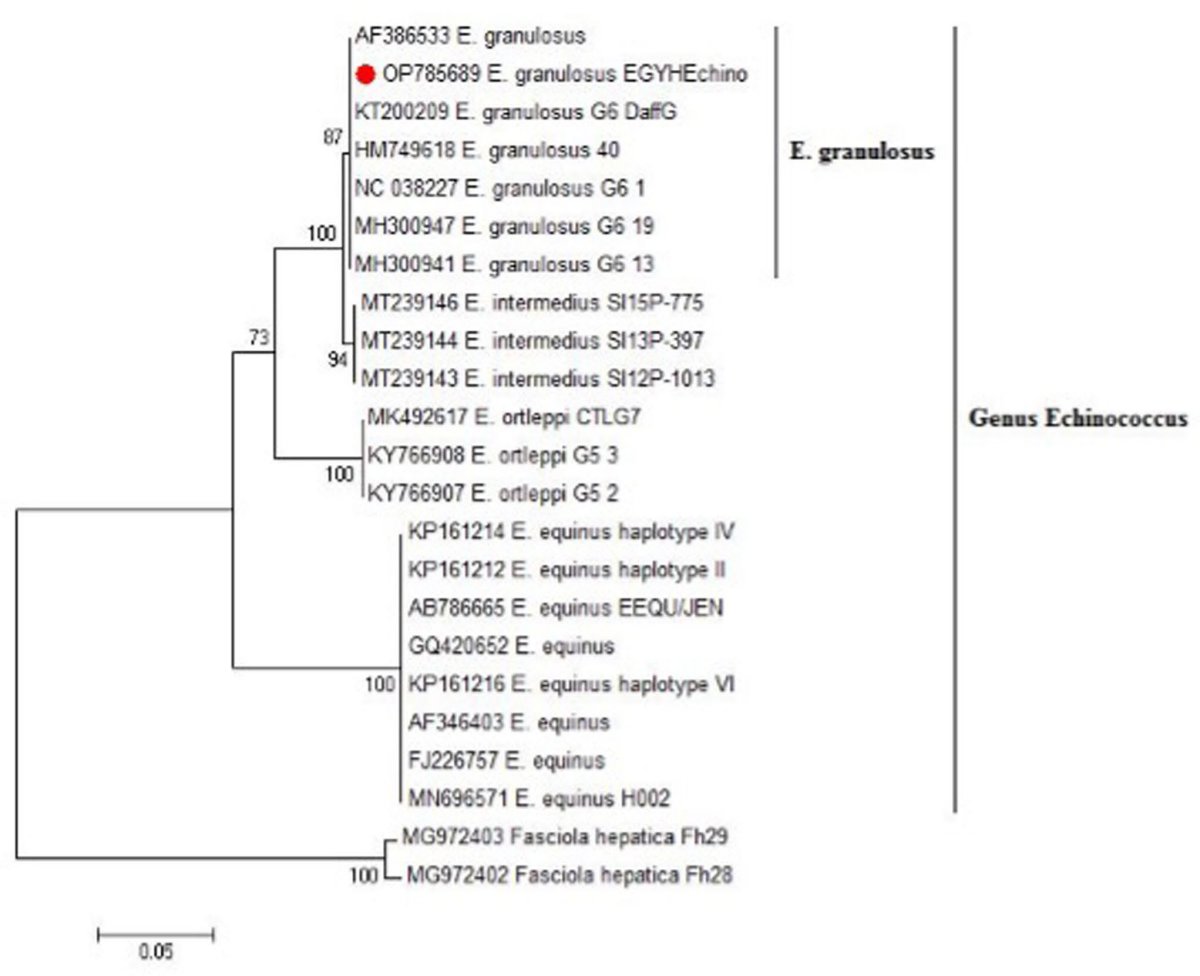
Phylogenetic analysis using the maximum likelihood method based on NAD1 gene for *Echinococcus* sp. Our obtained isolate is highlighted (red dot). There was a total of 459 positions in the final dataset. Our genotype clustered in a well-supported branch (bootstrap value 78) with other *E. granulosus* references. The scale bar represents a 5% nucleotide sequence divergence

Four DNA samples extracted from protoscoleces collected from human fertile liver cysts provided the expected amplicon size (~ 500 bp) for *Echinococcu*s spp. in the gel electrophoresis after PCR using NAD1 gene. Blast analysis showed the presence of genotype of *E. granulosus* in the investigated humans (GenBank: OP785689.1). In the phylogenetic tree, the resulted genotype clustered in a well-supported branch (bootstrap value 78) with other *E. granulosus* references **(**Fig. 1**).**

### SDS-Page

**Fig. 2:**
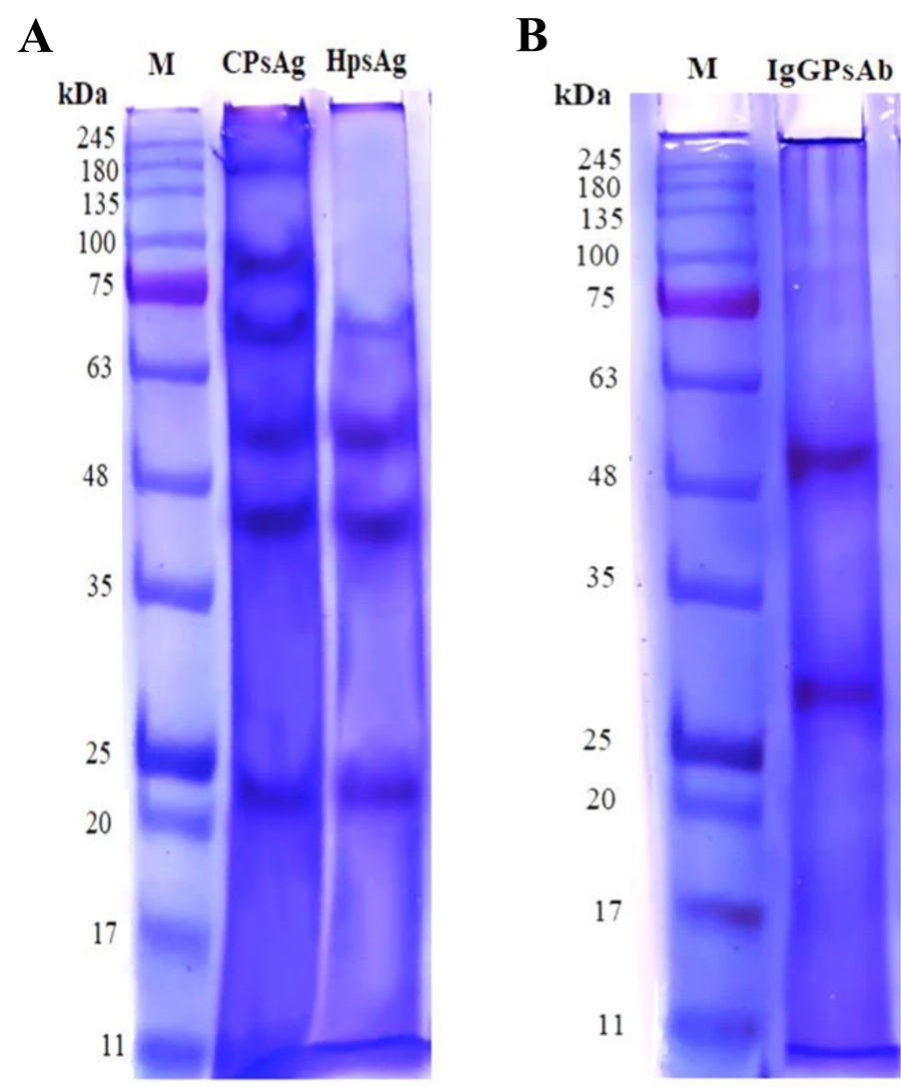
Electrophoresis profile of protoscoleces hydatid cyst antigens, 10% SDS-PAGE slab gel stained with Coomassie brilliant blue dye. **A**: Camel protoscoleces hydatid cyst antigens (CPsAg): Lane CPsAg and Human protoscoleces hydatid cyst antigen (HPsAg): Lane HPsAg. **B**: Purified IgG PsAb after precipitation with 50% ammonium sulfate and purified by Affinity chromatography on protein-A Sepharose gel column: Lane IgG PsAb. Molecular weight standard protein: Lane M

We assayed the purity of IgG PsAb, and the two prepared *E. granulosus* antigens CPsAg and HPsAg by SDS-PAGE. The results showed that the protein bands of prepared CPsAg appeared at different molecular weights: 180, 90, 68, 54, 42 and 22 kDa **(**Fig. 2A; **Lane CPsAg).** Whereas HPsAg shared in four common bands with CPsAg at molecular weights: 68, 54, 42, and 22 kDa **(**Fig. 2A; **Lane HPsAg).** The purified IgG PsAb had been resolved at two bands; the heavy chain band (H- chain band) was represented at 52 kDa, and at 32 kDa was the light chain band (L-chain band) **(**Fig. 2B; **Lane IgG PsAb).**

### The binding activities of purified IgG PsAb against CPsAg and HPsAg

**Fig. 3:**
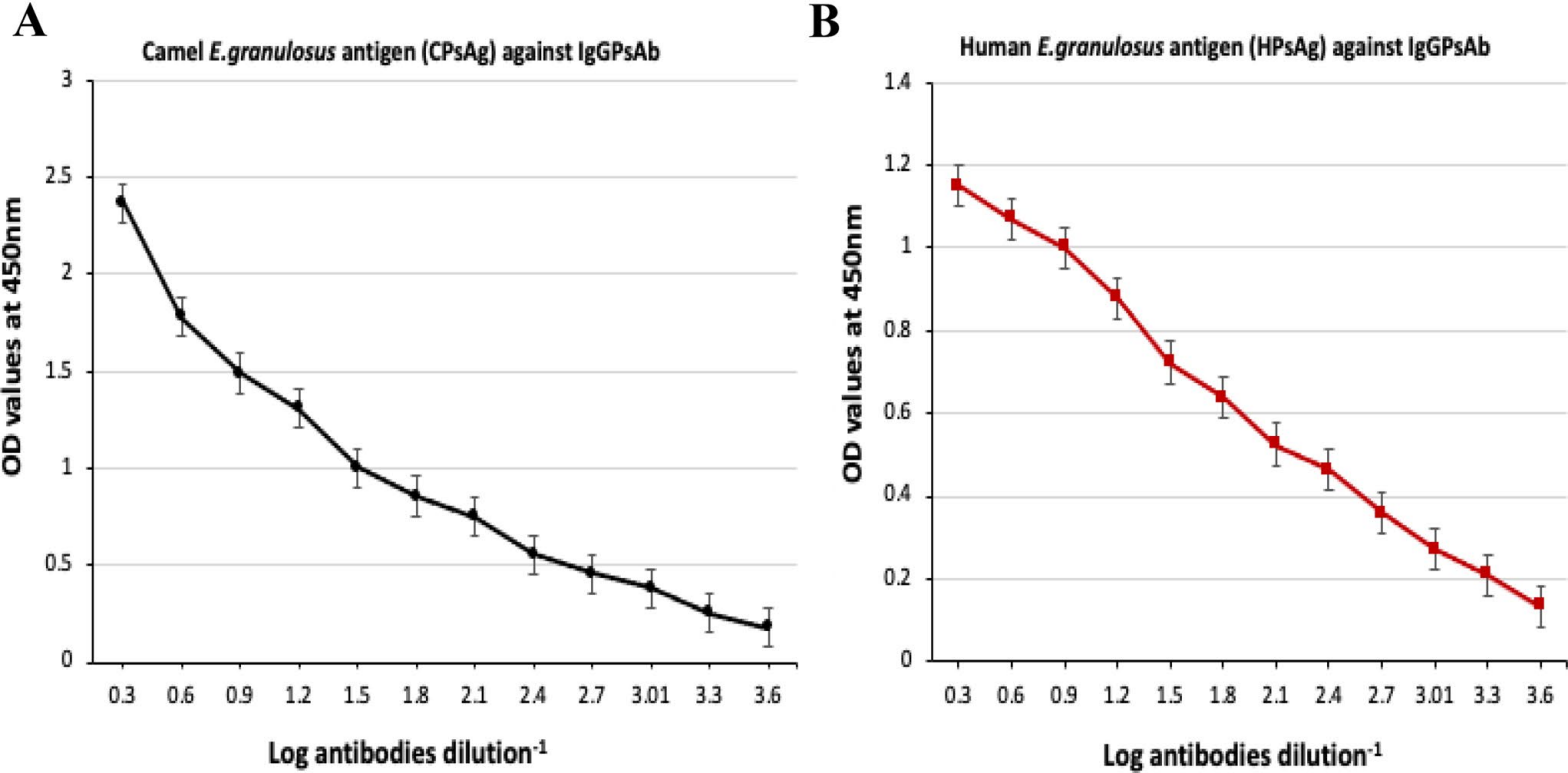
Binding activities in purified IgG PsAb towards camel *E. granulosus* antigen (CPsAg) **(A)** and Human *E. granulosus* antigen (HPsAg) **(B)** by indirect ELISA, at two-fold serially dilutions of specific purified IgG PsAb.

The success of developing antibodies in rabbits (IgG PsAb) was proved by indirect ELISA in which commercial Anti-rabbit IgG HRP-conjugate was used. The immune sera (IgG PsAb) were collected at high titers against antigens (CPsAg and HPsAg) of the antibodies with maximal OD of 1.8 and 1.2 respectively, at 1/100 dilution of IgG PsAb. The protein content in the crude rabbit serum containing anti-*E. granulosus* PsAb was 15.5 mg/ml. After purification the protein content of IgG PsAb was 12 mg/ml. As shown in Fig. 3, the purified IgG PsAb checked by two-fold serial dilutions reached to ~ 1: 4096 and still reacted positively with CPsAg **(**Fig. 3A**)** and HPsAg **(**Fig. 3B**).**

### Specificity of purified IgG PsAb in the diagnosis of CE

**Fig. 4:**
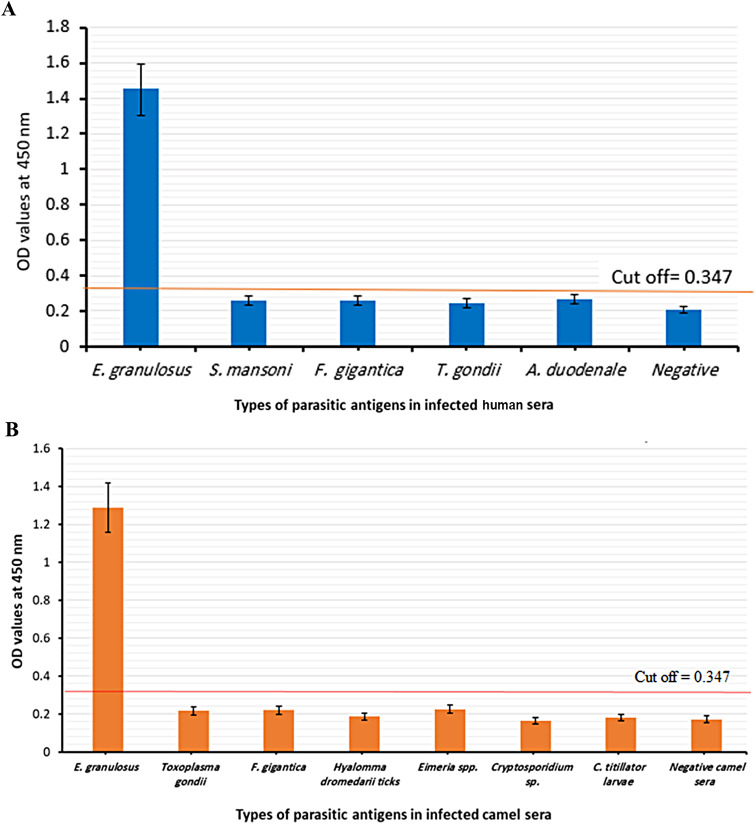
Detection of circulating *E. granulosus* antigen in sera of human **(A)** and camels **(B)** infected with *E. granulosus* or other parasites in comparison to negative controls sera by using Sandwich ELISA

Comparative evaluation of specificity of the purified IgG PsAb in detection circulating *E. granulosus* antigen in sera of human and serum of naturally infected camels with *E. granulosus* or other parasites in comparison to healthy controls for diagnosis of CE was determined by Sandwich ELISA as showed in Fig. 4A**&B**. The purified IgG PsAb showed strong reactivity against serum samples from human and camels infected with *E. granulosus* and no cross reactivity neither with free-healthy negative sera nor with others parasitic diseases (Human sera with Schistosomiasis, Fascioliasis, Toxoplasmosis, Ancylostomiasis and camel sera with Fascioliasis, ticks’ infestation, Eimeriosis, Cryptosporidiosis, Nasal myiasis, Toxoplasmosis). The cut-off value was 0.347. The mean OD value of CE infected camels was 1.288 ± 0.186 and it was 1.449 ± 0.172 in infected human sera which were both higher than negative control group (0.273 ± 0.198) and the other parasites groups (0.265 ± 0.025; Fig. 4A**&B**).

The sensitivity of the assay was calculated to be 98.25% sensitivity against human samples, where the sandwich ELISA detected circulating hydatid antigens in 56 cases out of 57 which were *E. granulosus* infected with surgical confirmation, ultrasound-proven, and presumptive diagnosis of CE. While 31 camel samples were detected as *E. granulosus* infected from 32 samples that confirmed by PM examination and the sensitivity of the assay was 96.9%. All the negative controls and positive samples for other parasites in human and camel samples were below the cut-off recording a 100% specificity in both human and camel as shown in Table 2.

**Table 2:** The sensitivity, specificity, PPV, NPV and diagnostic accuracy of sandwich ELISA for detection of *E. granulosus* antigen in human and camel serum

Host	Sensitivity	Specificity	PPV	NPV	Diagnostic Accuracy
Human	98.25%	100%	100%	98.24%	98.65%
Camel	96.9%	100%	100%	96.88%	97.87%

### Indirect ELISA

Antibody detection by indirect ELISA, using antigen CPsAg, showed that 96.5% of human serum samples (55 cases out of 57) had CE antibodies in their sera while cross reaction was noted in a few non-CE cases (16 negative CE out of 17 cases). A sensitivity of 96.5% and specificity of 94.1% was calculated with diagnostic accuracy 95.95%. While the sensitivity of the assay was 93.8% in camel serum samples (30 out of 32) were detected as *E. granulosus* infected and specificity was 93.3% (14 free from CE out of 15) with diagnostic accuracy 93.62% in camel sera (Table 3).

**Table 3:** The sensitivity, specificity, and diagnostic accuracy of Indirect ELISA for detection of *E. granulosus* antibodies in human and camel serum

Host	Sensitivity	Specificity	Diagnostic Accuracy
Human	96.5%	94.1%	95.95%
Camel	93.8%	93.3%	93.62%

### Detection of circulating hydatid antigen in random serum of human and camels for the diagnosis of CE by sandwich ELISA

**Fig. 5:**
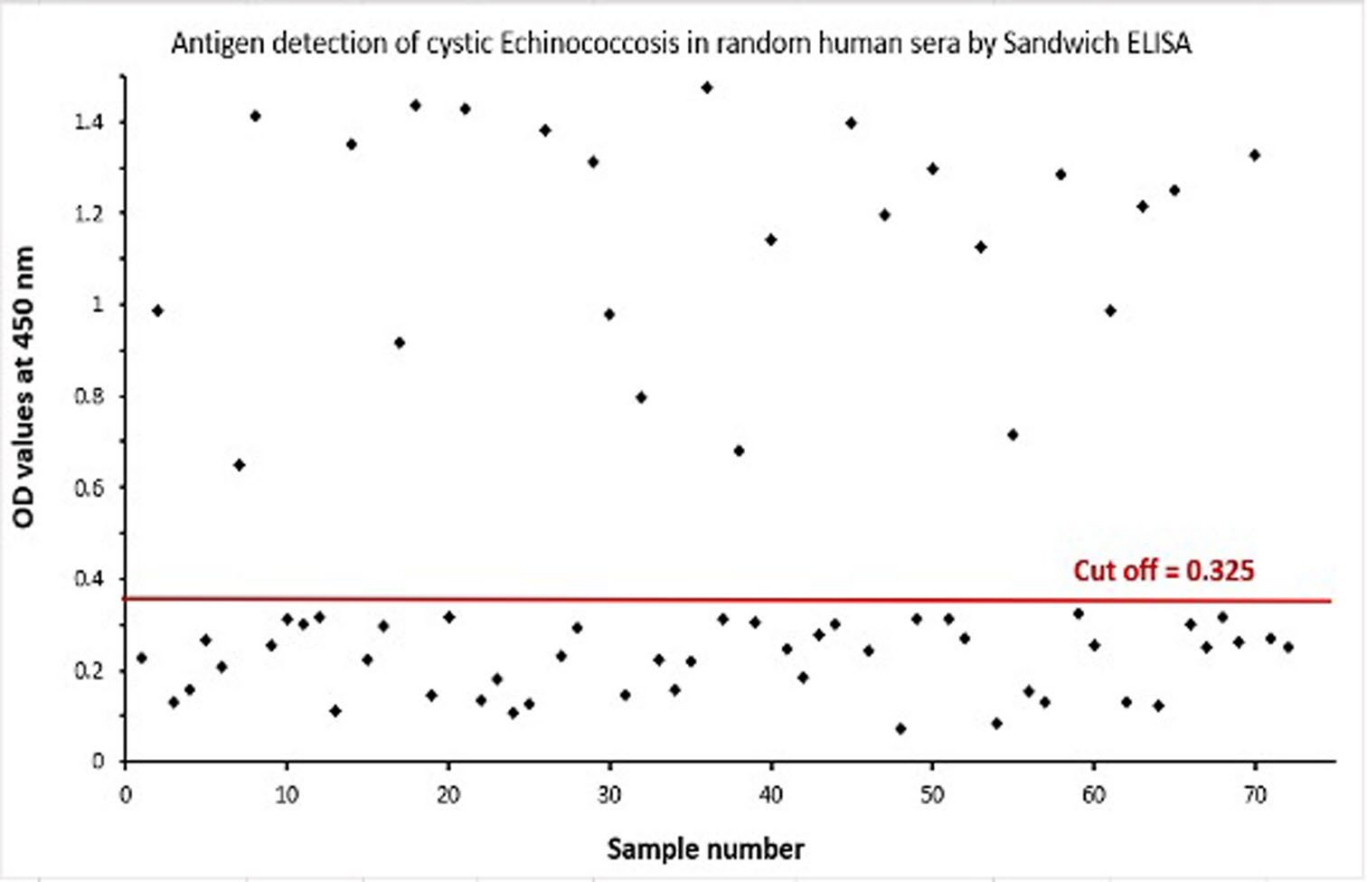
Detection of circulating *E. granulosus* antigen in random sera of human by Sandwich ELISA

**Fig. 6:**
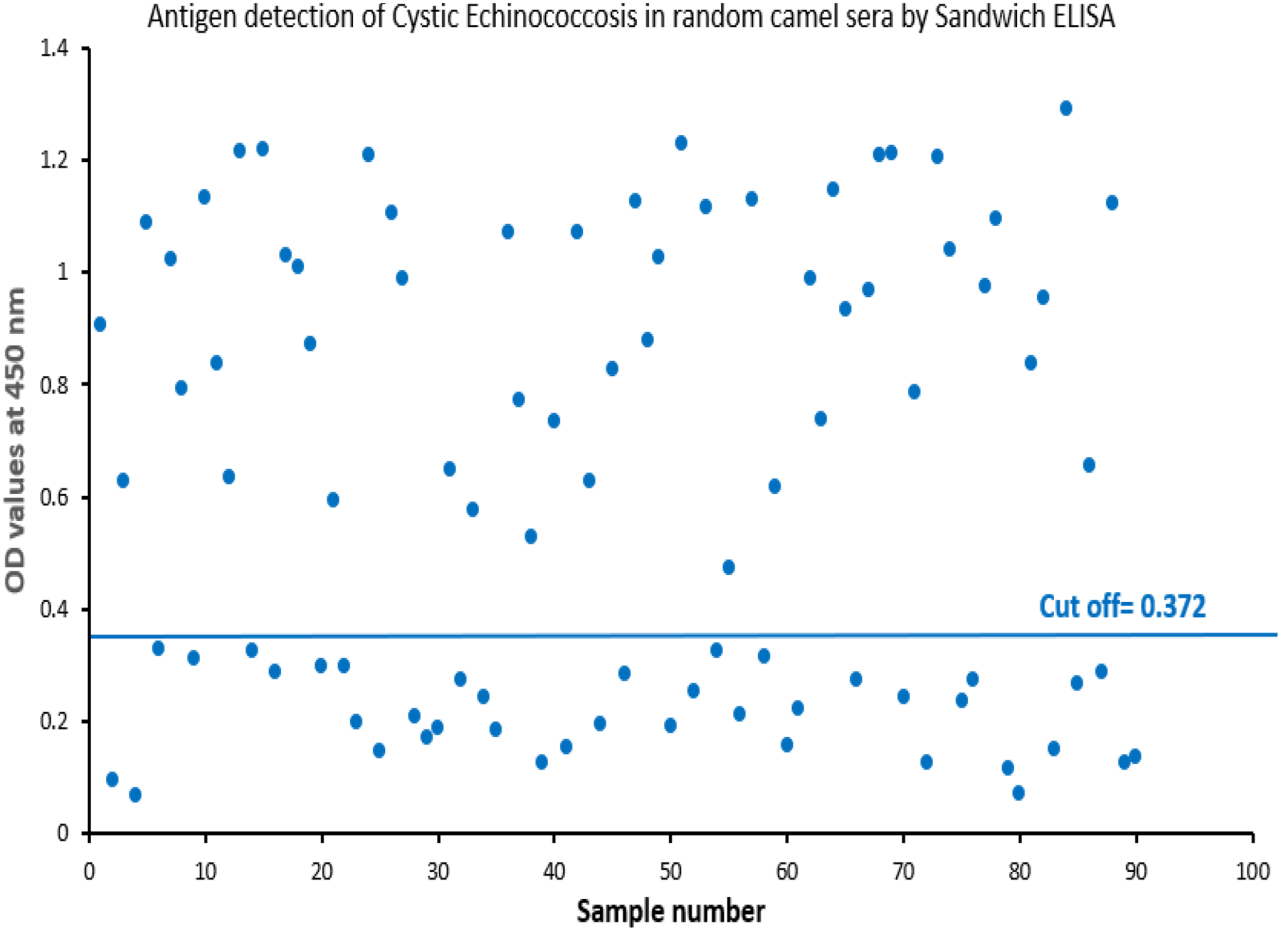
Detection of circulating *E. granulosus* antigen in random sera of camel by Sandwich ELISA

Using Sandwich ELISA, *E. granulosus* antigen was detected in 24 out of 72 (33.3%) of human samples (Fig. 5**)**. Whereas 50 infected camel samples were detected out of 90 random collected sample with a percentage of 55.6% (Fig. 6**)**.

## Discussion

CE is regarded as a significant public health problem in several countries, including Egypt **(**Hassanain et al. 2021; Hussien et al. 2022). In animals *E. granulosus* causes a high rate of lungs and liver condemnation in slaughtered domestic animals (Abo-Aziza et al. 2019). CE was recently included in the World Health Organization (WHO) strategy to combat neglected tropical diseases for its eradication or control **(**WHO 2020, 2021**).** CE is clinically detectable in humans through imaging techniques such as ultrasonography or radiology (Hussien et al. 2022), and after slaughtering in animals (Toaleb et al. 2023). The primary diagnosis should be confirmed by serological tests based on the detection of antibodies against *E. granulosus* antigens in serum (Dyab et al. 2017; Hassanain et al. 2021; Darabi et al. 2022). One of obstacles of this method is the difficulty of obtaining enough human *Echinococcus spp.* cysts from patients for antigen extraction and preparation. Therefore, most of the available serological assays depend on using antigens extracted from animal hydatid cysts to diagnose human CE **(**Bauomi et al. 2015; Hassanain et al. 2021). Also, commercially available serological kits mainly target HCF, which consists of both the host and parasite, its components vary from one cyst to another, which might lead to different HCF-based tests from one medical center to another (Pagnozzi et al. 2014; Ahn et al. 2015).

In the present study, we investigated the use of a purified anti-hydatid polyclonal antibody IgG (IgG CPsAb) for the detection circulating *E. granulosus* antigen in naturally infected human and camel sera using sandwich ELISA. We chose *E. granulosus* protoscoleces antigen which is shown to be a suitable diagnostic antigen candidate with high sensitivity and specificity in ELISA (Shahabinejad et al. 2020). We selected protoscoleces antigen separated from camel hydatid cysts (CPsAg), because the HCF antigen extracted from human hydatid cysts was unsuitable for diagnosis as it contains host protein like IgG which may cross react with specific IgG present in sera of CE infected human (Zhang et al. 2012). However, the immunological characterization of both camel and sheep hydatid fluid and protoscoleces antigens might be helpful for diagnosis of hydatidosis in human (Hassanain et al. 2016). When human and camel samples were surveyed using ELISA, the protoscoleces antigen was more efficient as a diagnostic antigen (Pagnozzi et al. 2016. Hassanain et al. (2021) found that protoscoleces antigen extracted from hydatid cysts of camel was the most potent antigen for diagnosing hydatidosis in humans and camels.

In the present study, most of *E. granulosus* cysts collected from human and camels were fertile containing large numbers of protoscoleces. The mean viability of the protoscoleces was (100%) in cysts removed from human during surgery and ranged from 90.9% in camels’ liver cysts to 58.3% in camels’ lung cysts. The fertility of the cysts and viability of protoscoleces demonstrate the perfect adaptation of the strain to human **(**Craig et al. 2007). The high rate of human hepatic infection reported in our study might be attributed to the fact that the liver acts as the primary filter in the human body and the lung is often thought to be the second filter **(**Xiao et al. 2005). A similar picture of organ affection has been reported by other studies as the most frequent site of hydatid cysts was the liver (50–70%) followed by the lung (20–30%) and less frequently, kidney, heart, bones **(**Wen et al. 2019; Şahin et al. 2023).

*Echinococcus sp.* isolate recovered from hydatid cyst protoscoleces of naturally infected humans in this study (Fig. 1; Genbank: OP785689.1) was identical to those of *E. granulosus sensu lato* genotype G6 detected in humans from Mongolia (GenBank: MH300971.1), camels from Iran (Genbank: HM749618.1 and NC_038227.1) and Mauritania (GenBank: MH300954.1 and MH300953.1) and sheep from Sudan (Genbank: MH300941.1 and MH300947.1). These results agreed with other previous reports, which assume that G6 is common in human and animals and is widespread in camel-raising countries **(**Romig et al. 2017). G6 was assumed to be the dominant genotype among *Echinococcus sp.* human isolates with a frequency of 58.1% **(**Abdelbaset et al. 2021). Also, hygienic and husbandry conditions of rearing animals, might increase transmission of *Echinococcus* spp. to humans **(**Casulli et al. 2022).

In the present study, CPsAg appeared at different molecular weights that were closely similar to bands (205, 71, and 68, 52, 41, and 22 kDa; Fig. 2) found in protoscoleces antigen previously reported by Hassanain et al. (2021). The purification procedures performed in our study were satisfactory and the purified IgG PsAb was characterized by two bands indicating that, the purified IgG PsAb appeared free from other proteins. This result agreed with El Deeb et al. (2017) who reported that the purity of anti-*E. granulosus* IgG PAb, assayed by 12.5% SDS-PAGE under reducing conditions, was represented by H- and L-chain bands at 50 and 31 kDa, respectively. In another study, Toaleb et al. (2023) investigated that the purified IgG germinal layer antibody was represented by four bands at 77, 65, 55 and 25 kDa. This difference might be due to the different method of preparation of crude antigens, different percentage of reagents of SDS-PAGE, rabbit immunization technique, and the method of polyclonal antibody IgG (IgG PsAb) preparation. In the present study, the purified IgG PsAb gave a strong reactivity to CPsAg and HPsAg as determined by indirect ELISA. Also, the purified IgG PsAb detected the circulating *E. granulosus* antigens in CE infected human sera and CE infected camel sera, when used as a primary capture to coat ELISA plates. Then IgG PsAb labeled with HRP-conjugate was captured again when sandwich ELISA was adopted using a pair of IgG PsAb against CPsAg, HPsAg antigens, anti- *E. granulosus* IgG PsAb and peroxidase-conjugated IgG polyclonal antibodies. After detection of *E. granulosus* circulating antigens by sandwich ELISA in human serum samples, all values equal to or above cut off values (0.347) were considered positive and 56 cases out of 57, which were infected by *E. granulosus*, gave positive results with 98.25% sensitivity and 96.9% (31 as *E. granulosus* infected from 32 positive samples) in infected camel serum samples. Specificity was 100% in healthy human and camel control serum samples with a diagnostic accuracy 98.65% of CE detection in human sera 97.87% in camel sera. These results showed higher sensitivity and specificity than other previously study in which the sensitivity was 90.48% and specificity was 91.3% when using sandwich ELISA with purified polyclonal antibody (PAb) raised against *E. granulosus* Cathepsin B for detection *E. granulosus* circulating antigen in human serum samples **(**El Deeb et al. 2017). In addition, it is higher than PAb conjugated with gold nanoparticles for detection *E. granulosus* antigen using nano-gold dot-ELISA, which recorded a sensitivity of 94.4% and a specificity of 90% with an accuracy value 92.9% of CE detection in human, camel, and sheep sera (Rashed et al. 2019). Moreover, in the present study, the in-house sandwich ELISA used to detect circulating CE antigen in human and camels’ sera was more sensitive and specific than other previous studies which used the purified anti-hydatid cyst fluid IgG using protein-A affinity chromatography which gave a sensitivity of 25.7% and a specificity of 98% (Sadjjadi et al. 2009). Furthermore, our results were more sensitive than the latex agglutination test (LAT) in the detection of circulating hydatid antigen in human serum which recorded a sensitivity of 72% and a specificity of 98% **(**Devi and Parija 2003**).** On other hand, when we compared our in-house sandwich ELISA based on polyclonal antibody IgG PsAb and the Indirect ELISA based on CPsAg (Fig. 3), we found that the indirect ELISA gave fewer positive samples in CE infected human and camels serum samples with lower sensitivity and specificity. These results might be related to lower antibody titer, which cannot be easily detected, in cerebral, ocular, and calcified cysts in human or some CE patients especially in old persons and infants **(**Ravinder et al. 1997). Also, the long persistence of anti-*E. granulosus* antibodies after surgical removal of the cysts results in unreliable diagnosis of relapse in patients **(**Todorov and Stojanov 1979**).**

The double antibody sandwich ELISA, used in our study, is a common method for measuring the presence and/or concentration of circulating parasite antigens (Zhang et al. 2012). Our results proved that the standard double antibody IgG PsAb sandwich ELISA could be a promising method for the detection of *E. granulosus* circulating antigens with high sensitivity and specificity. Indirect ELISA test conducted by Darabi et al. (2022) for detection of specific anti-hydatid cyst antibodies in human CE recorded a sensitivity of 95.2% and a and specificity of 96.8%, respectively, and also, sensitivity and specificity were higher than the of the commercial ELISA test which were recorded 96.8% (Darabi et al. 2022). Circulating hydatid antigens could be detected in serum only during active infection, and their levels decrease after surgical removal of hydatid cyst or successful chemotherapy (Devi and Parija 2003**).** In our study, sandwich ELISA used in the detection of circulating hydatid antigens in random collected samples from humans and camels giving infection rates of 33.3 and 55.6%, respectively (Figs. 5 and 6). Previous studies reported that hydatid cyst antigen could be detected in sera of 33–85% of hydatidosis patients (Gottstein 1984; Ravinder et al. 1997). Toaleb et al. (2023) found that infection rate was 48.7% in camels. High infection rates found in camels could explain the assumption that camels play a potential role in the maintaining of transmission cycle of hydatidosis **(**Gareh et al. 2021).

## Conclusion

The combination of clinical, radiological, and serological diagnosis is recommended in the diagnosis of hydatidosis. In our study, sandwich ELISA using IgG PsAb could offer a highly accurate, easy and low-cost assay for the diagnosis of CE in human and animals. In addition, the detection of antigen rather than antibodies could be a more reliable method for evaluating the status of infection which could be used for following up after medical or surgical treatment in humans and monitoring the efficacy of treatment in animals.

### Electronic supplementary material

Below is the link to the electronic supplementary material.

Supplementary Material 1

## Data Availability

No datasets were generated or analysed during the current study.

## Abbreviations

CE: Cystic Echinococcosis
AUC: Area Under Curve
CPsAg: Camel Protoscoleces Antigen
CT: Computed Tomography
*E*: *granulosus Echinococcus granulosus*
ELISA: Enzyme Linked Immunosorbent Assay
HCF: Hydatid Cyst Fluid
HPsAg: Human Protoscoleces Antigen
IgG: PsAb IgG Polyclonal Protoscoleces Antibodies
IHAT: Indirect Hemagglutination Test
LAT: Latex Agglutination Test
MRI: Magnetic Resonance Imaging
NAD1: NADH Dehydrogenase Subunit 1 gene
OD: Optical Density
OPD: Ortho-phenylenediamine
PAb: Polyclonal Antibody
PBS: Phosphate Buffered Saline
PCR: Polymerase Chain Reaction
PM: Post-Mortem
PsAg: Protoscoleces Antigen
ROC: Receiver Operating Characteristic
SD: Standard Deviation
SDS-PAGE: Sodium Dodecyl-Sulfate Polyacrylamide Gel Electrophoresis
WHO: World Health Organization
