# Supplementary figures and images for: Human and camel cystic echinococcosis – a polyclonal antibody-based sandwich ELISA for its serodiagnosis with molecular identification
Source: Vet Res Commun. 2024 Apr 26;48(4):2193–206. doi: 10.1007/s11259-024-10375-3 (PMC11315795; doi:10.1007/s11259-024-10375-3)

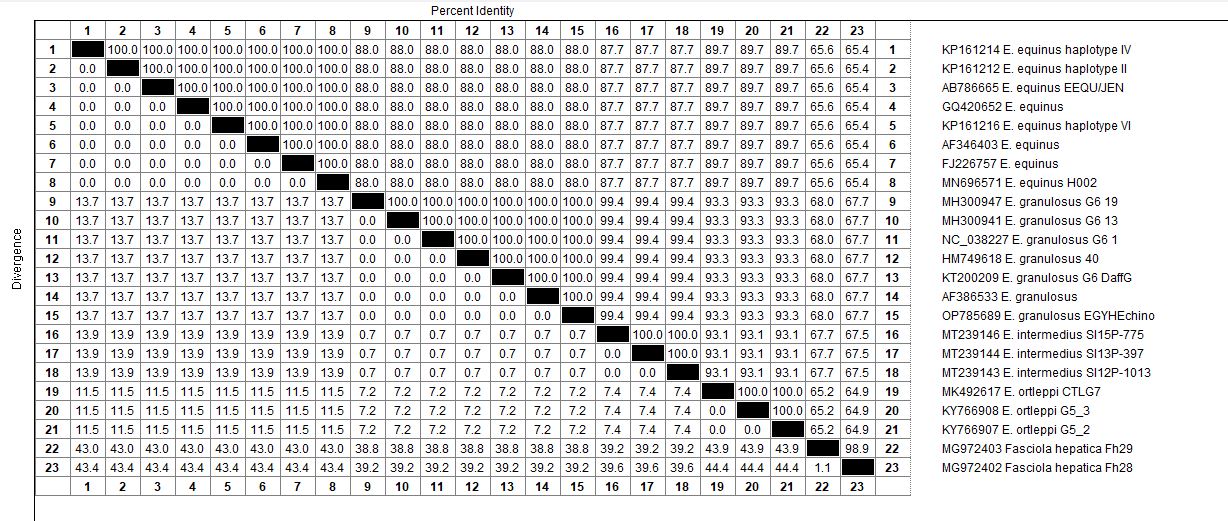

Supplement: Supplementary file 1 — Supplementary Material 1 [file 11259_2024_10375_MOESM1_ESM.jpg]
